# Prostate cancers in men under the age of 50: about a series in Togo, Sub-Saharan Africa

**DOI:** 10.1186/s12885-022-10425-6

**Published:** 2022-12-21

**Authors:** Tchin Darré, Toukilnan Djiwa, Tchilabalo Matchonna Kpatcha, Essodina Padja, Gado Napo-Koura, Tchin DARRE

**Affiliations:** 1 Department of Pathology, University Teaching Hospital of Lomé, Lomé, Togo; 2 Department of Urology, University Teaching Hospital of Lomé and Kara, Lomé, Togo; 3 Faculty of Health Sciences, University of Lomé, BP 1515, Lomé, Togo

**Keywords:** Cancer, Prostate, Young adult, Adenocarcinoma, Togo

## Abstract

**Background:**

Prostate cancer is a public health problem and increasingly diagnosed in men under 50 years of age. This cancer occurs much more in subjects of advanced age, generally over sixty. The aim of the study was to describe the epidemiological, clinical and histopathological aspects of prostate cancer in men under the age of 50 in Togo.

**Methods:**

It was a retrospective descriptive, cross-sectional study of histologically confirmed cases of prostate cancer in young adults at the Pathological Laboratory of Lomé over a period of 10 years (2011–2020).

**Results:**

In total, 29 cases of prostate cancer in patients under 50 years of age. The proportion of prostate cancers in men under 50 was 0.7% of all prostate cancers, The average age of the patients was 45 years with extremes of 35 and 49 years. Twelve patients had a family history of prostate cancer, with a statistically significant relationship between the existence of a family history of prostate cancer and the age of onset of the cancer (*p*-value = 0.03). The dominant clinical information was prostatic hypertrophy (40.37%), followed by acute urine retention (20.69%) and micturition disorders (17.27%). The median Prostate Specific Antigen (PSA) was 188 ng/ml with extremes of 20 ng/ml and 2100 ng/ml. A large proportion of patients had a PSA between 100 and 500 ng/ml. Histologically, they were all prostatic acinar adenocarcinomas. These adenocarcinomas were well differentiated (48%) and moderately differentiated (38%). The predominant histoprognostic grade was ISUP (International Society of Urological Pathology) grade 1 which was noted in 65.52%, followed by grade 2 in 20.69%.

**Conclusion:**

Prostate cancer in men under 50 years of age is relatively rare in Togo, sometimes occurring in the context of a family history of prostate cancer. Hence the importance of raising awareness among the male population, especially with a family history of prostate cancer, to start screening early, around the age of 40.

## Background

Prostate cancer, a public health problem, is defined as malignant cell proliferation at the expense of normal prostate constituents [1]. In 2020, prostate cancer was the 4th most diagnosed cancer in the world in terms of incidence, with 1.4 million new cases representing 7.3% of all diagnosed cancers and responsible for 375 thousand deaths [2]. Histologically, it is adenocarcinoma in more than 90% of cases; squamous cell carcinoma and neuroendocrine carcinoma are very rare [3, 4].

Prostate cancer mainly affects subjects over 70 years old with an average age at diagnosis of 74 years, diagnosed in 45% of cases after 75 years [2]. It is exceptional in men under 50, with an average frequency of 0.5% [5]. Young adult prostate cancer is a cancer that occurs in people up to the age of 50 [6, 7]. The incidence of prostate cancer in young adults (≤ 50 years old) increased 5.7 times, from 5.6 to 32 cases per 100,000 person-years between 1986 and 2008 [7, 8]. Using epidemiological surveillance results from 17 regions of the United States, Salinas et al. found an increase in the incidence of prostate cancer in men aged 20–49, especially since 1991; these cancers accounted for 10% of new cases diagnosed in 2013 [6]. Current literature suggests that the clinical features and prognosis of prostate cancer in young adults are contradictory and remain unresolved; some authors have suggested that young age is an indicator of poor prognosis [9, 10].

Several studies have reported a better survival outcome in men younger than 50 years [5, 11]. However, others have found no significant difference in recurrence, histologic grade, and stage of disease [12, 13].

Prostate cancer is the most common urological cancer in Togo representing 74.63% of all urological cancers; these cancers occur in 8.3% of subjects under 50 years old [3, 4].

As these studies do not focus specifically on prostate cancer in young adults, we initiated this study with the objective of updating the epidemiologic, clinical, and histopathologic aspects of prostate cancer in men under the age of 50.

## Methods

It was a cross-sectional descriptive study carried out among prostate cancers diagnosed histologically in subjects under 50 years of age at the Pathological Anatomy Laboratory of Lomé University Hospital over a period of 10 years (2011–2020). Togo is a country of 56,600Km2, with an estimated population of 7,200,000 inhabitants, located between Ghana in the west, Benin in the east and Burkina faso in th north. These cases were collected from the registers of the said laboratory. The study material consisted of biopsies and surgical specimens fixed in 10% buffered formalin and was processed according to conventional histology techniques.

The variables studied were frequency, age, personal or family history of prostate cancer, clinical signs, PSA level, nature of the specimen, histological group and type.

Data was entered twice in Microsoft Excel to reduce data entry errors and then exported to Epi Info version 7 software. A descriptive analysis was carried out with a view to highlighting the characteristics of the various qualitative and quantitative variables. We used the percentages for the qualitative variables and the means with their standard deviations for the quantitative variables. The statistical tests used were the Pearson Chi-square test for the qualitative variables and the Student test for the quantitative variables. The significance threshold was set at 0.05.

## Results

### Epidemiological data

A total, 29 cases of prostate cancer were collected in men under 50, an annual frequency of 2.9 cases. During the same period, 4200 cases of prostate cancer were globally collected. The proportion of prostate cancers in men under 50 was 0.7% of all prostate cancers. The average age of the subjects was 45 years with extremes of 35 and 49 years. Subjects between 45 and 50 years of age represented 48.83% (13 cases) of all cases. Regarding family history of prostate cancer, 12 patients (41.38%) had a family history of prostate cancer. We noted a statistically significant relationship between the existence of a family history of prostate cancer and the age of occurrence of the cancers (*p*-value = 0.03). (Table 1).

**Table 1:** Distribution of patients by family history of prostate cancer/age

	Age						
	**[30–35[**	**[35–40[**	**[40–45[**	**[45–50[**	**Total**	**%**	***P***-value
Family history of cancer							**0.03**
No	2	3	5	7	17	58.62	
Yes	0	1	5	6	12	41.38	
Total (%)	6.89	13.8	34.48	44.83	100		

### Clinical and biological data

Clinical information was represented by prostatic hypertrophy in 12 cases (41.38%), acute retention of urine in 6 cases (20.69%), micturition disorders without further specification in 5 cases (17.24%), hematuria in 3 cases (10.34%), back pain with 2 cases (6.90%) and adenopathy in 1 case (3.45%). The median PSA level was 188 ng/ml with extremes of 20 ng/ml and 2100 ng/ml. Eighteen patients (18; 62.07%) had a PSA level between 100 and 500 ng/ml. (Table 2)

**Table 2:** Distribution of patients by PSA level (ng/ml)

	Value	%
**< 100**	08	27.58
**[100–500[**	18	62.07
**[1000–1500[**	01	3.46
**> 1500**	02	6.89
**Total**	**29**	**100**

### Pathological data

The diagnosis was made on 19 prostate biopsies (65.52%) and 10 prostate adenomectomy specimens (34.48%). Histologically, all were prostatic acinar adenocarcinomas. These adenocarcinomas were well differentiated in 14 cases (48.27%), moderately differentiated in 11 cases (37.93%) and poorly differentiated in 4 cases (13.79%).

ISUP histoprognostic grade 1 (Gleason score 6) was found in 19 cases (65.52%), followed by grade 5 (Gleason score 9 or 10) in 4 cases (13.8%). There was no statistically significant relationship between the histoprognostic grade of ISUP and the age of cancer occurrence (*p*-value = 1.06), nor with the existence of a family history of cancer (*p*-value = 2.21). (Table 3).

**Table 3:** Distribution of patients by ISUP grade/age/family history of prostate cancer

	ISUP Grade					
	**Grade 1**	**Grade 2**	**Grade 3**	**Grade 5**	**Total**	***P***-value
**Age**						1.06
[30–35[	2	0	0	0	2	
[35–40[	3	0	1	0	4	
[40–45[	8	0	1	1	10	
[45–50[	6	3	1	3	13	
**Family history of cancer**						2.21
No	12	1	2	2	17	
Yes	7	2	1	2	12	
**Total (%)**	**65.52**	**10.34**	**10.34**	**13.8**	**100**	

For the 10 cases of adenomectomy, the pTNM stage was specified in 6 cases. These were 4 cases of stage pT1N0M0, one case of stage pT2N0M0 and pT2N1M0 respectively.

The surgical limits were in healthy areas in 9 cases (R0) and in microscopically tumorous areas in 1 case (R1).

## Discussion

In our study, the proportion of prostate cancer in subjects under 50 years of age was 0.7%. This proportion is similar to that of Alioune et al. in Senegal who found a frequency of 0.45% [14]. This frequency is lower than that of Catalona et al. who found a frequency of 2% of prostate cancer in subjects under 50 years of age [15]. The frequency of prostate cancer in young adults is 4% in Australians and Asians, and 9% and 3% respectively in Caucasians and black Americans [5]. This low frequency in sub-Saharan Africa is underestimated and may be related to the under-medicalization of the health care system, the geographical and financial inaccessibility of health care facilities, the difficulties of diagnosis, the shortage of specialist pathologists and oncologists, and the absence of a program to control cancer in general and prostate cancer in particular.

The average age of our subjects was 45 years. Similar mean ages have been reported by Alioune et al. and Varkarakis et al. of 44.99 and 41.7 years, respectively [14, 16].

It is estimated that 43% of prostate cancers occurring before the age of 50 are hereditary forms [10, 17]. In our series, 41.38% of patients had a family history of prostate cancer, with a statistically significant relationship between the existence of a family history of prostate cancer and the age of onset of the cancers (*p*-value = 0.03). Rouprêt et al. found a family history of prostate cancer in 33.6% of patients [18].

Clinically, 41.38% of our patients had prostatic hypertrophy. In the series of Alioune et al. and Huang et al. the discovery was incidental in the majority of cases, respectively in 55.9% and 38% [10, 14].

The median PSA level of our patients was 188 ng/ml. This level is much higher than that of Alioune et al. and Varkarakis, which were 26.62 ng/ml and 3.8 ng/ml respectively [14, 16]. Screening for prostate cancer in the presence of elevated serum PSA levels has been used in men over the age of 50 since at least 1992. No medical organization has recommended PSA screening for men in their 30 or 40 s [5].

Histologically, these were all prostatic acinar adenocarcinomas. These adenocarcinomas were predominantly ISUP grade 1 (Gleason score 6) in 65.52%, followed by ISUP grade 5 (Gleason score 9 or 10) in 13.8%. Varkarakis et al. and Huang et al. found a predominance of ISUP grade 2 [10, 16]. Alioune et al. found a predominance of ISUP grade 3 (Gleason score 7 = 4 + 3) [14]. Similar findings were made by Ji et al. who found a predominance of ISUP grade 3, with a statistically significant correlation with age (*p*-value = 0.002) [19]. Ji et al. report that men aged ≤ 50 years or > 75 years have clinically significant higher grades of prostate cancer compared to patients aged 55–75 years [19].

We did not find a statistically significant relationship between the histoprognostic grade of ISUP and age of cancer occurrence (*p*-value = 1.06), nor with the existence of a family history of prostate cancers (*p*-value = 2.21). Similarly, Huang et al. did not find a statistically significant association between histopronostic grade and age (*p*-value = 0.652) [10]. Bleyer et al. and Ji et al. found a statistically significant relationship between histopronostic grade and age with *p*-values of 0.043 and 0.002 respectively [5, 19]. Several studies have reported a better survival outcome in men younger than 50 years [5, 11]. However, others have found no significant difference in recurrence, histologic grade, and disease stage [12, 13]. This raises the issue of the age of onset of prostate cancer screening, especially with more and more cases diagnosed around 40 years old [20].

Regarding the pTNM classification, we found 4 cases of stage pT1N0M0 out of the 6 cases specified. Review of the literature indicates that earlier detection of the prostate cancer with low grade and stage disease in young men has a superior disease outcome [7].

Aprikian et *al.* revealed similar histologic grade and disease stage, between the younger and older population [21].

## Limitations

Our study has limitations common to retrospective studies, including the absence of data such as family history of prostate cancer in the majority of the records. The absence of a population-based cancer registry also makes it difficult to compare our results to those of the general population. However, it is of interest because it is the second study in sub-Saharan Africa on prostate cancer in subjects under 50 years of age, apart from the study by Alioune et al. [14].

## Conclusion

Prostate cancer is a public health problem in Togo, occurring most often in men over 75 years of age. These cancers of young adults are most often hereditary forms, diagnosed generally at high grades ; which is not the case in Togo, probably due to the small sample size. Hence the importance of raising awareness among the male population, especially with a family history of prostate cancer, to start screening early, around the age of 40.

## Data Availability

Extracted data are with the corresponding author and available under reasonable request.

## Abbreviations

PSA: Prostate Specific Antigen
ISUP: International Society of Urological Pathology
